# Bridging the Digital Healthcare Gap in Rural Areas to Strengthen Communities and Enhance Care Delivery

**DOI:** 10.7759/cureus.77922

**Published:** 2025-01-24

**Authors:** Ryuichi Ohta

**Affiliations:** 1 Community Care, Unnan City Hospital, Unnan, JPN

**Keywords:** family medicine, general medicine, health literacy, help-seeking behavior, rural, social media

## Abstract

This editorial explores the transformative impact of digital health technologies in rural areas, focusing on their role in bridging healthcare gaps and empowering communities. Drawing from research in Unnan City, the article highlights how social network-based platforms, such as the LINE app (LY Corporation, Tokyo, Japan), have enabled residents to seek medical advice and share health concerns anonymously. The discussion covers both the positive impacts and challenges, including misinformation and the digital divide, while emphasizing the need for thoughtful implementation and continuous education. The editorial calls for collaborative efforts to enhance the reliability and accessibility of digital health solutions, ensuring equitable healthcare access for all.

## Editorial

Have you ever wondered how modern technology could bridge the vast healthcare gap in our rural communities? Picture a grandmother in a secluded village, her nearest doctor miles away, now able to receive medical advice with a simple tap on her smartphone. This isn't just technological advancement; it's a revolution in accessibility and comfort for those who once felt left behind.

The importance of digital health

Health literacy is not just a buzzword; it's a vital lifeline. Understanding and effectively using health information can drastically improve one's quality of life [1]. Yet, in many rural areas, challenges like geographical isolation, limited infrastructure, and a shortage of medical professionals often put essential health services out of reach [2].

Imagine a world without these barriers. The internet has the potential to break down these walls, but there’s a catch: the digital divide [3]. How can we ensure that everyone, including the elderly and less tech-savvy, can navigate this new digital landscape without feeling overwhelmed or excluded?

From my experience

During my research in Unnan City, I was deeply moved by the stories of residents who, thanks to digital tools, experienced transformative changes in their approach to health care [4]. I recall meeting TK, a local farmer who initially doubted the efficacy of health apps. Over time, TK found that consulting with a doctor via his phone was not just convenient but empowering. He said, "I used to delay seeing a doctor because of the distance. Now, help is right at my fingertips."

Our study utilized a social network-based consulting system through the LINE app (LY Corporation, Tokyo, Japan), developed by family physicians at Unnan City Hospital [4]. This platform allowed residents to post health-related concerns anonymously, creating an environment of openness and trust. Though not a replacement for direct medical consultations, the platform has been a significant step forward in engaging communities traditionally limited by access to healthcare [4].

The impact of community and technology

What would your older patients think about using an app to manage their health? The feedback from our participants, especially the elderly, was eye-opening. They appreciated the anonymity and the ease of use, which removed the intimidation factor often associated with technology and medical consultations.

Despite its limitations, such as the lack of immediate responses, the platform fostered a sense of community and mutual support. Over 10,432 posts from 621 participating citizens underline a robust demand for such interfaces, revealing themes like mutual exploration for information, temporary collaborations for empathy-building, and creating a community of mutual assistance [4].

The dual nature of digital health platforms

While these platforms can enhance dialogue and support, they can also become grounds for misinformation and conflict. A thoughtful approach is necessary to address these challenges. This includes better moderation, reliable content validation, and continuous education for users and healthcare providers [5].

What now? The road ahead

The insights gained from this research illuminate the current use and challenges of digital health technologies in rural settings and pave the way for innovative solutions tailored to these unique needs [4]. As someone who finds immense satisfaction in conducting qualitative research, the findings from this study surprised and inspired me. They showed that integrating digital tools in healthcare, especially in under-resourced rural areas, is no longer just a convenience but a necessity.

As we look to the future, how can we enhance the reliability of these digital solutions, foster responsible usage, and integrate them seamlessly with traditional healthcare services? This integration aims to reduce the healthcare disparity between rural and urban areas, ensuring equitable access to quality healthcare for all.

Let's be part of the change

So, how will you contribute to narrowing the healthcare access gap with digital tools? Whether you are a healthcare provider, a tech developer, or someone interested in making a difference, your actions can help transform rural healthcare landscapes, making them more accessible, responsive, and comprehensive. Let's not just be observers of change; let's drive it.

Join the conversation and tell us how digital health could fit into your clinical setting or community. Together, we can ensure that digital health tools do not create new barriers but break them down, enhancing equity of access for everyone.
